# Levels and related factors of occupational stress among nurses: hospital-based evidence from China, 2023

**DOI:** 10.3389/fpsyg.2024.1471640

**Published:** 2025-01-17

**Authors:** Xiaoying Zhong, Yan Zeng, Lin Peng, Xixi Li, Yuanli Jia, Changqing Pan, Bangjun Wang

**Affiliations:** ^1^Gynecology and Obstetrics, Mianyang Central Hospital, School of Medicine, University of Electronic Science and Technology of China, Mianyang, China; ^2^Department of Nursing, Mianyang Central Hospital, School of Medicine, University of Electronic Science and Technology of China, Mianyang, China

**Keywords:** occupational stress, nurse, low fertility, China, dysfunction

## Abstract

**Background:**

China’s birth rate continues to decline, reaching only 6.39% in 2023. In light of this trend, hospitals may need to reassess their allocation of resources, including funding, staffing, and facilities. Nurses may face job insecurity and uncertainty regarding their roles, which could prompt some to consider transitioning to different specialties. This study aimed to investigate the levels of occupational stress among nurses in the context of low fertility in China in 2023 and to identify the factors contributing to this stress. In addition, the study sought to explore the relationship between family dysfunction, low fertility rates, and occupational stress levels.

**Methods:**

This descriptive cross-sectional study involved 270 nurses working in hospitals, who were recruited between December 2023 and January 2024 through a Chinese free web-based platform (Sojump) to complete online questionnaires. In addition to demographic information, the Nursing Job Stressors Scale (NJSS) and the Family APGAR Index were utilized for data collection. The data were analyzed using descriptive and inferential statistics, including correlation and multiple linear regression analysis. For continuous variables, the mean, standard deviation (SD), median, and interquartile range were reported, while counts and percentages were used for categorical variables. The independent *t*-test and one-way analysis of variance were employed for univariate analysis. Multiple linear regression was utilized for multivariate analysis. A *p*-value of less than 0.05 was considered statistically significant.

**Results:**

The participants’ average scores for the NJSS and Family APGAR Index were 1.76 ± 0.58 and 6.35 ± 3.30 points, respectively. In addition, workload and time pressure were rated highest among the sub-scales of the NJSS. The top five job stressors for nurses were Q3 (*Wages and other benefits are low*), Q1 (*The social status of nursing is too low*), Q5 (*Frequent shift work*), Q12 (*Too much useless paperwork*), and Q16 (*Fear of mistakes and accidents at work*). The score of the Family APGAR Index demonstrated a negative correlation with occupational stress (*r* = −0.19, *p* < 0.001). The results of the multiple linear regression analysis showed that a high level of worry about losing one’s job (SE = 0.044, *β* = 0.152, *t* = 2.567, *p* = 0.011) and poorer family APGAR scores (SE = 0.035, *β* = −0.202, *t* = −3.406, *p* < 0.001) were associated with higher NJSS scores.

**Conclusion:**

The nurses reported experiencing a moderate level of occupational stress in the context of low fertility in China. The key predictors of occupational stress among the nurses included concerns about job security and the Family APGAR classification. Implementing fair compensation and providing more effective family-oriented support programs are essential for reducing occupational stress among nurses.

## 1 Introduction

### 1.1 Background

Occupational stress refers to the response that individuals may experience when confronted with work demands and pressures that exceed their knowledge and abilities, thereby challenging their capacity to cope (de Wijn et al., 2022; Zaghini et al., 2020). Occupation-related stress is a growing concern worldwide (Xu et al., 2024). Research findings indicate that occupational stress adversely affects individuals’ psychological and physical health, contributes to burnout, and impacts organizational effectiveness (Alinejad et al., 2023; Kabakleh et al., 2020; Kowalczuk et al., 2023; Li et al., 2021; Yang et al., 2021; Zaghini et al., 2020).

Nursing is one of the most stressful and high-risk professions (Norful et al., 2024). Nurses face a range of occupational risks, including infections, unsafe patient handling, hazardous chemicals, radiation, psychosocial hazards, violence, harassment, injuries, and issues related to marital satisfaction (Adib-Hajbaghery et al., 2021; Babapour et al., 2022; Ekingen et al., 2023; Martin et al., 2023). Approximately 16.2 to 53.5% of nurses were reported to have latent tuberculosis, a prevalence that is 25 times higher than that of the general population (Aldhawyan et al., 2024; Johansen et al., 2023; Kinikar et al., 2019; Severo et al., 2011). Between 61 and 75.9% of nurses in clinical settings reported experiencing chronic lower back pain, compared to only 18% among office workers (Fujii et al., 2019; Sun et al., 2021). Globally, between 33.1 and 46% of nurses reported experiencing some form of violence in the workplace (Bagnasco et al., 2024; Li et al., 2024; McLaughlin and Khemthong, 2024). During the COVID-19 pandemic, approximately 56% of nurses experienced depression, while between 39 and 42.4% reported experiencing anxiety (Al-Amer et al., 2022; Pang et al., 2021). Furthermore, nurses are reportedly at a higher risk of suicide across all regions of the world (Hofstetter and Mayer, 2022; Kramer et al., 2024).

The occupational stress experienced by nurses follows universal patterns but also presents unique challenges across different cultural and healthcare contexts (Yuan and Fang, 2024). A study conducted in Uganda found that 70.5% of nurses reported experiencing chronic stress (Kabunga et al., 2023). In China, 64.71% of physicians and nurses in emergency departments considered their occupational stress to be high or very high after contracting COVID-19 (Lv et al., 2023). A cross-sectional study in Iran indicated (Ghaderi et al., 2024) that nurses experienced a higher-than-average level of occupational stress during the COVID-19 pandemic. In Ethiopia, 47.8% of nurses working in public hospitals reported experiencing occupational stress. In the context of the stress process model, various factors may influence the level of occupational stress among nurses, including personal, work-related, and social factors (Turner, 2013). Regarding the personal characteristics of nurses, a cross-sectional study conducted in Iran demonstrated that emotional and moral intelligence mediated the relationship between occupational stress and job performance among nurses (Alinejad et al., 2023). Previous studies (Chen et al., 2022; Meneguin et al., 2024; Wu et al., 2023; Yao et al., 2022; Zhou et al., 2022) have found that psychological wellbeing is negatively associated with occupational stress in pediatric nurses. According to a study in Iran (Werke and Weret, 2023), the presence of children was significantly linked to job stress. In terms of work-related factors, studies in China and Korea (Choi and Kim, 2022; Yuan and Fang, 2024) indicated that a higher frequency of night shifts, extended work hours, and insufficient rest time were associated with increased occupational stress levels. In addition, a study in Brazil (Meneguin et al., 2024) found that the number of hospitals where nurses have employment ties and their relationships with management were associated with stress levels among nurses. A study in China (Yang et al., 2021) revealed that occupational stress was influenced by distributive justice among nurses in public hospitals. Regarding the social factors, numerous studies (Kasidouli et al., 2024; Kowalczuk et al., 2023; Şanlıtürk, 2021; Saravanan et al., 2023) have shown a high prevalence of occupational stress among health workers during the COVID-19 pandemic.

China’s fertility rate has significantly declined, dropping to approximately 1.3 children per woman in 2022, which is well below the replacement level of 2.1 (Zhai and Jin, 2023). In 2023, the birth rate reached a record low of only 6.39%, marking the lowest since the establishment of the country (Yang et al., 2024; Zhao et al., 2023). This decline in fertility directly impacts the number of births, resulting in fewer obstetric patients and a subsequent reduction in demand for pediatric services. Consequently, hospitals may need to reevaluate their resource allocation, including funding, staffing, and facilities. Nurses, particularly those in obstetrics and pediatrics, may experience job insecurity or uncertainty regarding their roles, which could lead them to consider transitioning to different specialties. This situation could result in higher turnover rates and a potential need to seek employment in other fields, thereby affecting workforce stability.

Despite the extensive literature on the levels and related factors of occupational stress among nurses, no studies have investigated this issue in the context of low fertility in China. Nurses are the backbone of any functioning healthcare system; therefore, this study was conducted to examine the levels and related factors of occupational stress among nurses in the context of low fertility in China in 2023.

## 2 Materials and methods

### 2.1 Research design

This study utilized a cross-sectional design and was conducted through an online survey. The Strengthening the Reporting of Observational Studies in Epidemiology (STROBE) checklist was followed in reporting the findings of the study.

### 2.2 Research participants

Participants were recruited using convenience sampling. Although convenience sampling has its limitations, researchers can identify key demographics, ensure diversity, and broaden the geographical scope to enhance sample representativeness and generalizability.

The inclusion criteria were as follows: (a) nurses aged 18 to 60 years as nurses in this age range represent a vital segment of the healthcare workforce, contributing to patient care, education, and leadership within the field; (b) registered nurse; and (c) nurses with the ability to accurately read and write in Chinese. Student nurses and those undergoing standardized training were excluded.

### 2.3 Research tool

#### 2.3.1 Demographic characteristics questionnaire

The demographic characteristics questionnaire included basic information about the nurses, such as age, education, marital status, monthly income, major, position, years of working experience, department, night shifts in the past three months, hospital level, teaching hospital, hospital type, nursing manager, participation in advanced studies/specialist nurse training, employment form, status of children, and concerns about job security.

#### 2.3.2 The nursing job stressors scale (NJSS)

The Nursing Job Stressors Scale (NJSS) is a self-administered, multidimensional scale designed to assess the levels of job stress among nurses (Sun et al., 2017). This tool consists of 35 items divided into five domains: professional and career issues (1–7 items), workload and time pressure (8–12 items), resource and environment problems (13–15 items), patient care and interaction (16–26 items), and interpersonal relationships and management problems (27–35 items) (Luan et al., 2017; Yuan and Fang, 2024). Each item is rated on a 4-point Likert-type scale (1–4), with the total score ranging from 35 to 140. The internal consistency of the NJSS was reported as 0.931 in China (Sun et al., 2017). In the present study, the Cronbach’s alpha for the total NJSS was 0.968, indicating that the internal consistency of the measure was adequate.

#### 2.3.3 The Family APGAR Index

The Family APGAR Index is composed of five items. Each item is rated on a 3-point Likert-type scale (0–2), with the total score ranging from 0 to 10 (Babincak and Kacmarova, 2023). Based on the total score, the Family APGAR Index categorizes families as severely dysfunctional (0–3), moderately dysfunctional (4–6), or functional (7–10) (Castilla et al., 2014). In China, the Family APGAR Index was reported to have acceptable internal consistency (*α* = 0.942) (Zhang et al., 2020). In the current study, the Cronbach’s alpha for the Family APGAR Index was 0.936, indicating that the internal consistency of this tool was adequate.

### 2.4 Collection of data

The data for this study were collected between December 2023 and January 2024 through a free Chinese web-based platform (Sojump) for administering online questionnaires. (1) *Platform selection*: The surveys were distributed using the Sojump platform, which is a widely recognized online survey tool known for its user-friendly interface and robust features. This platform allows for the easy creation, distribution, and analysis of surveys. (2) *Target population:* The target population for the survey included registered nurses, specifically those aged 18 to 60 years. (3) *Social media invitations and outreach:* We sent personalized two-dimensional code invitations to nursing associations, hospitals, and healthcare organizations, encouraging them to share the survey link with their members. We utilized social media platforms (e.g., WeChat and QQ) to promote the survey, targeting nursing groups and forums to increase visibility and encourage participation.

### 2.5 Quality control strategies

To ensure the data quality and validity of the online responses, we used various approaches.

#### 2.5.1 The first approach was survey collection

Anonymity and confidentiality. The participants were assured that their responses would remain anonymous and confidential. This was communicated clearly in the survey introduction to encourage honest and accurate responses.

#### 2.5.2 The second approach was to ensure data quality and validity

*(1) Pre-testing:* The survey instrument was pre-tested with 20 nurses prior to full distribution. This helped identify any ambiguities in the questions and allowed for adjustments to improve clarity and relevance. *(2) Use of established scales:* Where applicable, we incorporated established and validated scales. This enhanced the reliability of the data collected. *(3) Response Time Analysis:* We analyzed the average time taken to complete the survey to identify any outliers or instances of incomplete responses. The participants who completed the questionnaire in less than 60 s were eliminated during the final data-cleaning session. *(4) Screening for inconsistencies:* After data collection, we implemented checks for inconsistent or contradictory responses. For instance, if the participants indicated extreme values across related questions, we flagged these for further review. *(5) Address tracking*: Sojump allows for IP address tracking, which helped us identify and exclude duplicate responses from the same source.

### 2.6 Analysis of data

Data were analyzed using SPSS 29.0 software with both descriptive and inferential statistics.

- Mean and standard deviation (SD) were reported for continuous variables that followed a normal distribution, while median and interquartile were reported for continuous variables that followed a non-normal distribution.
- Number and percentage were reported for categorical variables.
- In accordance with the demographic characteristics, the differences in occupational stress were analyzed using the independent *t*-test and one-way analysis of variance.
- The correlation between the Family APGAR Index and occupational stress was analyzed using Pearson’s correlation coefficients.
- Multiple linear regression was performed to evaluate the statistical significance of the effect of the demographic factors and the Family APGAR Index on occupational stress among the nurses using the stepwise method.
- A *p*-value of <0.05 was considered statistically significant.

### 2.7 Ethical considerations

The study protocols received endorsement from the Bio-ethical Commission of the Mianyang Central Hospital, under reference no. 2023KY082.

## 3 Research results

### 3.1 The demographic characteristics of the participants

In this study, 47.8% of the participants were aged between 18 and 30 years, and 65.9% had a university degree. A total of 69.7% of the participants were married. Moreover, 55.2% of the participants had a monthly income between 3,001 and 6,000 yuan. Other demographic characteristics of the participants are displayed in Table 1.

**Table 1:** Occupational stress in accordance with the demographic characteristics of the participants (*N* = 270).

Variables	*n* (%)	Occupational stress			
		M ± SD (Raw score)	M ± SD (Standardized score)	Test type	*p*-value
Age (years)					
18–30	129 (47.8)	62.12 ± 21.26	1.78 ± 0.61	*F* = 0.346^b^	0.708
31–40	102 (37.8)	60.30 ± 17.81	1.72 ± 0.51		
≥ 41	39 (14.4)	62.97 ± 21.89	1.80 ± 0.63		
Education					
Secondary technical school	7 (2.6)	71.14 ± 23.31	2.03 ± 0.67	*F* = 0.984^b^	0.401
Junior college	81 (30.0)	63.28 ± 22.79	1.81 ± 0.65		
University	178 (65.9)	60.52 ± 18.66	1.73 ± 0.53		
Graduate school or higher	4 (1.5)	56.25 ± 16.07	1.61 ± 0.46		
Marital status					
Unmarried	74 (27.4)	61.61 ± 21.43	1.76 ± 0.61	*F* = 0.004^b^	0.996
Married	187 (69.3)	61.57 ± 19.57	1.76 ± 0.56		
Divorce	9 (3.3)	61.0 ± 21.46	1.74 ± 0.61		
Monthly income (Yuan)					
≤3,000	26 (9.6)	76.42 ± 23.26	2.18 ± 0.66	*F* = 5.558^b^	0.001**
3,001–6,000	149 (55.2)	59.94 ± 19.62	1.71 ± 0.56		
6,001–10,000	91 (33.7)	60.16 ± 18.49	1.72 ± 0.53		
≥10,001	4 (1.5)	57.0 ± 15.64	1.63 ± 0.45		
Major					
Nurse	196 (72.6)	61.32 ± 20.78	1.75 ± 0.59	*t* = −0.316^a^	0.752
Midwife	74 (27.4)	62.19 ± 18.19	1.78 ± 0.52		
Position					
Junior nurse	154 (57.0)	61.25 ± 20.42	1.75 ± 0.58	*F* = 0.048^b^	0.953
Intermediate nurse	93 (34.5)	62.08 ± 19.22	1.77 ± 0.55		
Senior nurse	23 (8.5)	61.52 ± 21.91	1.76 ± 0.63		
Years of working experience					
≤2	37 (13.7)	58.49 ± 20.07	1.67 ± 0.57	*F* = 1.111^b^	0.352
3–5	45 (16.7)	63.44 ± 22.63	1.81 ± 0.65		
6–10	65 (24.0)	63.68 ± 21.33	1.82 ± 0.61		
11–20	88 (32.6)	58.98 ± 16.71	1.69 ± 0.48		
≥21	35 (13.0)	64.94 ± 21.82	1.86 ± 0.62		
Working department					
Department of pediatrics	15 (5.6)	63.07 ± 20.66	1.80 ± 0.59	*F* = 0.407^b^	0.666
Gynecology and obstetrics	181 (67.0)	60.78 ± 18.48	1.74 ± 0.53		
Other	74 (27.4)	63.15 ± 23.59	1.80 ± 0.67		
Night shifts in the past three months (days)					
0–6	112 (41.5)	60.64 ± 20.72	1.73 ± 0.59	*F* = 0.251^b^	0.860
7–12	22 (8.1)	61.50 ± 15.38	1.76 ± 0.44		
13–24	62 (23.0)	63.40 ± 21.43	1.81 ± 0.61		
≥25	74 (27.4)	61.42 ± 19.40	1.75 ± 0.55		
Hospital level					
Level III	181 (67.0)	61.48 ± 19.70	1.76 ± 0.56	*F* = 0.224^b^	0.800
Level II	44 (16.3)	63.16 ± 18.27	1.80 ± 0.52		
Level I	45 (16.7)	60.33 ± 23.35	1.72 ± 0.67		
Teaching hospital					
Yes	196 (72.6)	61.33 ± 19.18	1.75 ± 0.55	*t* = −0.282^a^	0.778
No	74 (27.4)	62.16 ± 22.41	1.78 ± 0.64		
Hospital type					
General hospital	238 (88.1)	61.59 ± 19.69	1.76 ± 0.56	*t* = 0.065^a^	0.949
Specialized hospitals	32 (11.9)	61.34 ± 23.07	1.75 ± 0.66		
Nursing manager					
Yes	49 (18.1)	62.27 ± 19.98	1.75 ± 0.58	*t* = −0.272^a^	0.786
No	221 (81.9)	61.40 ± 20.40	1.78 ± 0.57		
Engaging in advanced studies/Specialist nurse training					
Yes	91 (33.7)	62.19 ± 19.81	1.78 ± 0.57	*t* = 0.366^a^	0.715
No	179 (66.3)	61.24 ± 20.26	1.75 ± 0.58		
Employment form					
Labor contract	221 (81.9)	61.14 ± 19.96	1.75 ± 0.57	*t* = −0.728^a^	0.467
Career establishment	49 (18.1)	63.45 ± 20.67	1.81 ± 0.59		
Status of children					
Yes	174 (64.4)	62.64 ± 19.27	1.79 ± 0.55	*t* = 1.19^a^	0.235
No	96 (35.6)	59.60 ± 21.43	1.70 ± 0.61		
Worry about losing my job					
Never	62 (23.0)	62.21 ± 22.86	1.78 ± 0.65	*F* = 6.872^b^	<0.001**
Rarely	85 (31.5)	56.78 ± 17.09	1.62 ± 0.49		
Sometimes	106 (39.3)	61.49 ± 17.88	1.76 ± 0.51		
Always	9 (3.2)	84.33 ± 16.93	2.41 ± 0.48		
All the time	8 (3.0)	82.63 ± 29.19	2.36 ± 0.83		
The classification of the Family APGAR Index					
Severely dysfunctional family	51 (18.9)	63.21 ± 19.09	1.81 ± 0.55	*F* = 14.590^b^	<0.001**
Moderately dysfunctional family	90 (33.3)	69.47 ± 19.92	1.98 ± 0.57		
Functional family	129 (47.8)	55.39 ± 18.58	1.58 ± 0.53		

M, Mean; SD, Standard deviation. ^a^the independent *t*-test; ^b^one-way analysis of variance. **p ≤ 0.001.

### 3.2 Levels of occupational stress and the Family APGAR Index of the participants

The participants’ average scores for the NJSS and Family APGAR Index were 1.76 ± 0.58 and 6.35 ± 3.30 points, respectively (Table 2).

**Table 2:** Descriptive statistics of the study variables (*N* = 270).

Variables	Raw score				Standardized score		
	Range	Min	Max	M ± SD	Min	Max	M ± SD
Nursing Job Stressors Scale							
Professional and Career Issues	7–28	7	28	13.67 ± 4.80	1	4	1.95 ± 0.69
Workload and Time Pressure	5–20	5	20	9.87 ± 3.84	1	4	1.97 ± 0.77
Resource and Environment Problems	3–12	3	11	4.69 ± 1.96	1	3.67	1.56 ± 0.65
Patient Care and Interaction	11–44	11	44	19.44 ± 7.23	1	4	1.77 ± 0.66
Interpersonal Relationships and Management Problems	8–32	9	30	13.88 ± 5.30	1	3.33	1.54 ± 0.59
Total scale	35–140	35	131	61.56 ± 20.08	1	3.74	1.76 ± 0.58
Family APGAR Index							
Adaptability	0–2	0	2	1.23 ± 0.75	—		
Cooperation	0–2	0	2	1.18 ± 0.75			
Development	0–2	0	2	1.29 ± 0.74			
Affection	0–2	0	2	1.31 ± 0.73			
Problem-Solving Capacity	0–2	0	2	1.34 ± 0.73			
Total scale	0–10	0	10	6.35 ± 3.30			

Workload and time pressure were rated highest among the sub-scales of the NJSS. The top five job stressors for the nurses were Q3 (*Wages and other benefits are low*), Q1 (*The social status of nursing is too low*), Q5 (*Frequent shift work*), Q12 (*Too much useless paperwork*), and Q16 (*Fear of mistakes and accidents at work*).

### 3.3 Correlation between occupational stress and the Family APGAR Index of the participants

The relationship between the Family APGAR Index and occupational stress is an important area of study, particularly in understanding how family dynamics can influence individual wellbeing in the workplace. A negative correlation between these two variables was found in the present study, meaning that as scores on the Family APGAR Index increased (indicating better family functioning and support), occupational stress levels tended to decrease, and vice versa. By fostering supportive family environments, individuals may find themselves better equipped to manage occupational challenges, leading to healthier work experiences and overall wellbeing. The total NJSS score was negatively correlated with the Family APGAR Index (*r* = −0.19, *p* < 0.001) (Table 3).

**Table 3:** Correlations between the Nursing Job Stressors Scale and Family APGAR Index (*N* = 270).

Variables	1	2	3	4	5	6	7
1. Family APGAR Index	1						
2. Professional and Career Issues	−0.28**	1					
3. Workload and Time Pressure	−0.15*	0.67**	1				
4. Resource and Environment Problems	−0.14*	0.58**	0.65**	1			
5. Patient Care and Interaction	−0.12	0.64**	0.68**	0.66**	1		
6. Interpersonal Relationships and Management Problems	−0.15*	0.67**	0.65**	0.64**	0.79**	1	
7. Nursing Job Stressor Scale	−0.19**	0.83**	0.83**	0.77**	0.92**	0.90**	1

*p ≤ 0.05; **p ≤ 0.001.

### 3.4 Influential factors of occupational stress in the participants

The multiple linear regression analysis revealed that a high level of worry about losing one’s job and poorer classification of the Family APGAR scores were associated with higher scores on the NJSS (adjusted *R*^2^ = 0.061, *F* = 29.357, *p* < 0.001).

The regression equation was as follows:


$$\begin{array}{l} y=1.895+0.152x_{1}-2.202x_{\begin{array}{l} 2 \end{array}}\\ \mathrm{Note}:y=\mathrm{score}\;\mathrm{of}\;\mathrm{NJSS};x_{1}=\mathrm{level}\;\mathrm{of}\;\mathrm{worried}\;\mathrm{about}\;\mathrm{losing}\;\mathrm{my}\;\mathrm{job};\\ x_{2}=\mathrm{classification}\;\mathrm{of}\;\;\mathrm{Family}\;\mathrm{APGAR} \end{array}$$

## 4 Discussion

Low fertility rates in China can significantly affect the occupational stress levels of nurses, a crucial part of the healthcare workforce. This dynamic is particularly important to understand in the context of China’s unique demographic and healthcare challenges. Low birth rates can have a significant impact on staffing and workload imbalances, resource allocation, and organizational changes (Song et al., 2018). On the one hand, a decrease in the number of births may reduce demand for maternal services and maternity and neonatal care services, potentially leading to job insecurity (Bolan et al., 2021). On the other hand, low birth rates may prompt healthcare facilities to implement budget cuts, reducing resources available for nursing staff, such as training, support services, and staffing levels (Aranda et al., 2022). Hence, with fewer births, nurses may experience concerns about job security, leading to heightened stress levels (Table 4).

**Table 4:** Results of the multiple linear regression analysis of the factors related to occupational stress in the nurses (*N* = 270).

Variables	*B*	SE	*β*	*t-*value	*p*-value
Constant	1.895	0.139	-	13.589	<0.001
Level of worry about losing my job	−0.151	0.044	0.152	2.567	0.011
Classification of the Family APGAR Index	0.091	0.035	−0.202	−3.406	<0.001

B, Unstandardized Coefficient; SE, Standard Error; *β*, Standardized Coefficient. R^2^ = 0.068, adjusted R^2^ = 0.061, *F* = 29.357, *p* < 0.001.

The current study aimed to investigate the levels and related factors of occupational stress among nurses in the context of low fertility in China. The findings showed that the mean total score of occupational stress was 61.56 ± 20.08 (standardized score = 1.76 ± 0.58), indicating a moderate level of occupational stress among the nurses in this context. A previous study (Zhang et al., 2024) reported that the total score of the NJSS was 2.56 ± 0.47 for nurses caring for patients with gynecological cancer. The level of occupational stress among the nurses was slightly lower compared to the finding of Zhang et al. (2024). This might be attributed to the potentially high levels of job stress in the oncology department, such as prolonged exposure to negative emotions and chemotherapy drugs (Sarıbudak and Üstün, 2024).

Workload and time pressure were rated highest among the sub-scales of the NJSS, which is in line with the findings of Yuan and Fang (2024). The study pointed out that extended work hours and insufficient rest time are linked to increased levels of occupational stress (Yuan and Fang, 2024). Addressing these issues often requires a combination of individual strategies (such as time management and setting boundaries) and organizational support (such as promoting work-life balance and reducing excessive workloads) (Panahi et al., 2022).

The findings of the study by Wei et al. (2023) on the surgical system in China reported that the top job stressor for nurses in the operating room was ‘*Wages and other benefits are low*’ (Wei et al., 2023). The result of the present study reported that the top five job stressors for the nurses were ‘*Wages and other benefits are low*, *The social status of nursing is too low*, *Frequent shift work*, *Too much useless paperwork*, and *Fear of mistakes and accidents at work*. The first and fifth stressors are consistent with the findings of Wei et al. (2023) study. The study by Meneguin et al. (2024) in Brazil also found a significant correlation between occupational stress levels among nurses and income (Meneguin et al., 2024). A national survey of 9,256 psychiatric nurses in China reported that 92.5% of the participants desired an income increase of at least 10%, with more than half expressing dissatisfaction with their income (Gu et al., 2024). Fair compensation can motivate healthcare professionals to deliver high-quality care and improve patient outcomes (Carter et al., 2016).

Time pressure, lack of control over work tasks, long working hours, shift work, lack of support, and moral injury were reported as important risk factors for occupational stress among health nurses (Meneguin et al., 2024). We found that the nurses who were more worried about losing their jobs perceived higher levels of occupational stress. Without changes, the closure, diversion, and unemployment in obstetrics, which have often been reported in the Chinese scientific community and popular media (Chen, 2024), may lead to the collapse of obstetrics (Chen, 2024). Therefore, managers can reduce occupational stress among nurses by focusing on effective strategies related to hospital transformation (Af Ugglas et al., 2020; Barnett et al., 2018).

A good family environment can improve nurses’ understanding of stressful events (Ma et al., 2023). The results of the current study demonstrated that occupational stress was higher in nurses with poorer classification of the Family APGAR scores. This correlation suggests that the quality of family support may play a significant role in influencing occupational stress among nurses.

Low birth rates significantly impact the occupational stress levels of nurses in China, affecting their workload, job security, quality of care, and overall wellbeing. As the Chinese healthcare system navigates these challenges, it is essential to recognize the implications of low birth rates on nursing staff and implement strategies that support their mental health, job satisfaction, and retention. By addressing these issues, healthcare leaders can ensure that the nursing workforce remains resilient and capable of meeting the healthcare demands of the population, even as demographic trends evolve. Enhancing compensation and providing family-oriented support specifically for nurses are crucial, given the demanding nature of their work and the challenges they face in balancing professional and personal responsibilities (Pailhé and Solaz, 2019). Competitive salary adjustments and enhanced family-oriented support for nurses are among the necessary strategies to address occupational stress among nurses. Firstly, salaries must be regularly reviewed and adjusted based on industry standards, geographical cost of living, and the unique demands of nursing roles (Ravari et al., 2020). Offering salaries that reflect the high level of skill and responsibility involved in nursing can improve retention. Secondly, flexible shift options should be provided to help nurses balance their professional duties with family obligations (Alkhawaldeh et al., 2020). Thirdly, access to employee assistance programs (EAPs) that offer counseling and resources for nurses dealing with family issues and stress management must be provided, along with mental health support (Panahi et al., 2022).

There are some limitations in this study. Firstly, 270 nurses were primarily recruited through convenience sampling from southwest China. This might have led to sampling bias and not be representative of nurses in other regions. The second limitation is subjectivity with self-reported measures. The NJSS and the Family APGAR Index, as self-reported measures, were used to collect data in the current study. Hence, the results relied on the individuals’ perceptions, feelings, and memories, which could vary widely. While convenience sampling is inherently limited, researchers can strive to include a more diverse range of participants within the available sample to enhance representativeness. Furthermore, future research could use multiple methods of data collection (e.g., qualitative interviews and observational studies) to corroborate findings and provide a more comprehensive understanding of occupational stress among nurses.

## 5 Conclusion

In conclusion, this study showed that the nurses experienced a moderate level of occupational stress in the context of low fertility in China. The key predictors of occupational stress among the nurses were the level of worry about losing their jobs and poorer classification of the Family APGAR scores. The current findings may be useful for implementing fair compensation and providing more effective family-oriented support programs for nurses.

## Data Availability

The datasets presented in this study can be found in online repositories. The names of the repository/repositories and accession number(s) can be found in the article/supplementary material.
